# Chimeric degraders in neurodegenerative diseases: Challenges and future directions

**DOI:** 10.1515/jtim-2025-0099

**Published:** 2025-12-22

**Authors:** Hanyuan Zheng, Kun Cui, Jian Mao, You Wan, Jie Zheng

**Affiliations:** Beijing Life Science Academy, Beijing, China; Neuroscience Research Institute and Department of Neurobiology, School of Basic Medical Sciences, Peking University, Key Laboratory for Neuroscience, Ministry of Education/National Health Commission, Peking University, Beijing, China

Neurodegenerative disorders, including Alzheimer’s, Parkinson’s, and Huntington’s disease, *etc*., are characterized by the accumulation of misfolded, aggregation-prone proteins such as hyperphosphorylated tau, α-synuclein, and mutant huntingtin, which disrupt neuronal function and ultimately lead to cell death.^[1,2]^ Conventional therapies, such as small-molecule inhibitors and antibodies, have achieved limited success against these “undruggable” proteins.^[3]^

In recent years, targeted protein degradation (TPD) has emerged as a strategy to eliminate disease-driving proteins by co-opting endogenous disposal systems.^[4]^ These agents, collectively termed targeting chimeras (TACs), are bifunctional molecules that use one warhead to bind the target protein and a second module to recruit a cellular degradation mechanism. The prototypical proteolysis-targeting chimeras (PROTACs) link a target-binding ligand to an E3 ubiquitin ligase, thereby triggering the ubiquitination of the target and subsequent degradation through the proteasomal pathway. Several PROTACs have entered oncology clinical trials, and proof-of-concept studies indicate that proteins implicated in neurodegeneration can also be targeted.

Beyond PROTACs, an expanding repertoire has emerged—autophagy-targeting chimera (AUTACs) direct targets, including large protein aggregates, to macroautophagy for lysosomal degradation.^[5]^ Lysosome-targeting chimera (LYTACs) label extracellular or membrane proteins for endocytosis and lysosomal disposal.^[6]^ Dephosphorylation-targeting chimeras (DEPTACs) recruit phosphatases to reverse tau hyperphosphorylation, which facilitates downstream clearance.^[7]^ Although early results are encouraging, critical challenges must be addressed before chimeric degraders can realize their therapeutic potential in neurodegenerative disease. This perspective outlines three principal hurdles and potential solutions (Figure 1).

## Pathological specificity and off-target toxicity

A central challenge is to ensure that TACs selectively engage pathological protein species while sparing physiological counterparts. Many proteins implicated in neurodegeneration, such as tau and α-synuclein, perform essential functions in neurons; indiscriminate degradation risks on-target toxicity. For example, tau stabilizes microtubules in healthy neurons, and wholesale depletion could compromise synaptic architecture unless the TAC preferentially recognizes misfolded, aggregated, or hyperphosphorylated tau. Likewise, because α-synuclein supports synaptic vesicle trafficking, TACs should target misfolded or aggregated α-synuclein that accumulates in Parkinson’s disease while preserving the soluble pool required for normal function.

Off-target effects can also arise when the warhead lacks sufficient selectivity or when the recruited E3 ligase brings the complex into proximity with unrelated proteins. At high concentrations, some PROTACs induce a “hook effect”, causing bystander ubiquitination and unintended degradation.^[8]^ Moreover, many PROTACs recruit the E3 ligase cereblon (CRBN), and thalidomide-based CRBN ligands can degrade endogenous CRBN substrates, potentially leading to immune or hematologic adverse effects.^[9]^ These examples underscore the need for exceptional specificity in TAC design for chronic neurological indications.

**Figure 1:**
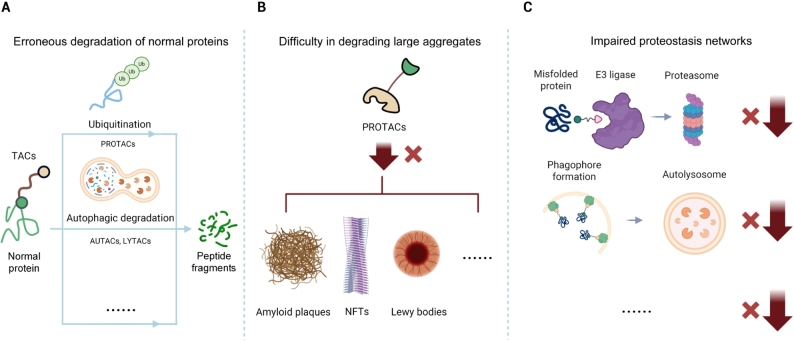
Key Challenges of Targeted Protein Degraders in Neurodegenerative Diseases. (A) Erroneous degradation of normal proteins. Some TACs, including PROTACs and related chimeric degraders, may lack disease specificity, potentially ubiquitinating or degrading normal proteins through autophagy or other pathways, which can lead to on-target toxicity. (B) Difficulty in degrading large aggregates. PROTACs that rely on the ubiquitin–proteasome system struggle to degrade large, insoluble protein aggregates, such as amyloid plaques, NFTs, and Lewy bodies. (C) Impaired proteostasis networks. Neurodegenerative disease cells often exhibit proteostasis dysregulation, with damaged UPS and autophagy-lysosome pathways that can weaken the efficacy of TACs such as PROTACs, AUTACs, LYTACs, and related modalities, necessitating strategies to bypass or restore proteostasis. TACs: termed targeting chimeras; PROTACs: prototypical proteolysis-targeting chimeras; NFTs: neurofibrillary tangles; UPS: ubiquitin-proteasome system; AUTACs: autophagy-targeting chimera; LYTACs: Lysosome-targeting chimera.

Advances in TACs engineering are beginning to address this challenge. One strategy is to design ligands that recognize structural features unique to pathogenic conformations or modifications, which are absent from the normal protein. The DEPTAC concept is up-and-coming. By recruiting phosphatases to hyperphosphorylated tau, pathological tau can be selectively dephosphorylated and subsequently cleared, whereas normal tau—with far fewer disease-linked phosphate groups—remains largely unaffected. The recently described D20 peptide DEPTAC exemplifies this approach, which markedly reduced neurotoxic tau in cellular and murine models with minimal impact on normal cells.^[10]^ Compared with traditional kinase inhibitors or global phosphatase activators, DEPTACs provide spatially and temporally confined dephosphorylation of selected substrates, which may translate into improved specificity and safety profiles in the central nervous system.

Beyond optimizing the warhead, enhancing context specificity is a desirable approach. Most current PROTACs recruit CRBN or von Hippel-Lindau (VHL), which are ubiquitously expressed. Recruiting an E3 ligase that is predominantly active in the affected brain region or neuronal subtype would confine TACs’ activity to sites where it is needed. The human genome encodes hundreds of E3 ligases, yet only a few have been leveraged to date. Tripartite motif-containing (TRIM) family members such as TRIM9 and TRIM67 are largely neuron-restricted and could concentrate activity within the central nervous system while reducing peripheral toxicity.^[11]^ However, unlike CRBN and VHL, these neuron-enriched ligases currently lack well-validated, drug-like small-molecule ligands, so discovering suitable binders remains a major frontier for degrader chemistry.

## Degrading insoluble protein aggregates

Advanced neurodegenerative disease is characterized by insoluble protein aggregates, including intraneuronal neurofibrillary tangles (NFTs) in Alzheimer’s disease, Lewy bodies containing α-synuclein in Parkinson’s disease, and huntingtin inclusions in Huntington’s disease. These aggregates are intrinsically difficult to degrade. The ubiquitin-proteasome system (UPS), on which PROTACs rely, is optimized for soluble proteins and small oligomers; large fibrillar assemblies are poor proteasome substrates because they cannot be efficiently unfolded and translocated into the proteasome core. Simply appending ubiquitin to massive aggregates is insufficient, and such substrates may persist or even overwhelm the UPS. Consequently, proteasome-based TACs alone may be ineffective once high-molecular-weight deposits have formed.

To address this limitation, investigators are leveraging autophagy, the cell’s other major degradation pathway. Autophagy engulfs large structures, including protein aggregates, damaged organelles, and intracellular pathogens, by sequestering them within autophagosomes that subsequently fuse with lysosomes. New TAC classes exploit this machinery. AUTACs append degradative tags that route targets to autophagy. Autophagosome-tethering compounds (ATTECs) and related molecules directly tether targets or aggregates to autophagy components, such as microtubule-associated protein 1 light chain 3 or sequestosome-1, thereby escorting them into nascent autophagosomes for lysosomal turnover.^[12]^ Because most aggregates are largely proteasome-resistant, autophagy-engaging strategies may be particularly effective. Several studies of tau-directed TACs suggest that activating autophagic clearance markedly reduces insoluble tau.

Looking ahead, incorporating autophagy-targeting mechanisms into new TAC design will likely be essential for disorders marked by extensive aggregation. A staged approach is conceivable: in early disease, proteasome-recruiting PROTACs could continuously remove misfolded monomers, whereas at later stages, autophagy-tethering TACs may be required to eliminate established deposits. Alternatively, a two-step regimen could first deploy a disaggregator to fragment fibrils, followed by PROTACs to degrade the resultant soluble species. In all cases, careful dose control is required to prevent inadvertent cytotoxicity, because excessive or dysregulated autophagy can be detrimental. In late disease stages, where autophagic flux is already compromised, further driving selective autophagy with TACs could saturate the pathway or interfere with other autophagy-dependent physiological processes, potentially disrupting global proteostasis. These safety considerations suggest a cautious approach to titration and a thorough preclinical assessment of long-term autophagy engagement.

## Impaired proteostasis in diseased neurons

Neurons affected by neurodegenerative disease not only accumulate toxic proteins but also exhibit compromised protein-quality-control systems. By the time pathology becomes evident, key components of proteostasis—within both the UPS and autophagy pathways—are often impaired or overloaded. In Parkinson’s disease, mutations in the Parkin E3 ligase and other ubiquitin-pathway genes reduce ubiquitination capacity. In Alzheimer’s disease and related tauopathies, proteasome activity is decreased and tau-relevant E3 ligases are altered, indicating UPS dysfunction; autophagy defects are likewise common.^[13]^ This situation presents a paradox for TAC therapies: how can TACs function if the pathways they recruit are themselves compromised?

One solution is to prime or augment degradative capacity in conjunction with TAC treatment. Combination regimens might modestly enhance proteasome activity or activate autophagy, thereby restoring baseline proteostasis before or during TAC administration. Such global enhancement must be carefully controlled: too little leaves aggregates unresolved, whereas excessive activation risks degradation of essential proteins or organelles. The objective is to reestablish near-physiological proteostasis rather than to drive wholesale catabolism.

Another strategy is to design TACs that circumvent fragile nodes within the patient’s proteostasis network. Because most PROTACs rely on CRBN or VHL, dysfunction of these ligases can blunt efficacy. Expanding the E3-ligase repertoire—particularly to brain-enriched or stress-inducible ligases that remain active in degenerating neurons—may bypass such bottlenecks. Furthermore, aligning the chosen degradation pathway with the cellular state is crucial: when proteasome function is severely impaired, autophagy-based TACs may be preferable, whereas if lysosomal or autophagic flux is blocked, proteasome-targeted approaches for soluble substrates could be more effective. In the longer term, biomarkers that report the relative status of the UPS and autophagy in individual patients may help stratify who is most likely to benefit from PROTAC- versus AUTAC-type interventions. Such stratification could integrate fluid or imaging markers of proteasome activity, lysosomal function, or aggregate load, aligning TAC selection with the principles of precision medicine.

## Conclusion

Chimeric degraders offer a transformative therapeutic modality for neurodegenerative diseases by directly eliminating proteins that drive pathology. The field has advanced rapidly from early PROTACs that degrade tau or huntingtin in cells to autophagy-based TACs and phosphatase-recruiting DEPTACs, and beyond. The key challenges—achieving high pathological specificity, clearing large aggregates, and operating in proteostasis-impaired cells—are substantial but tractable. A further hurdle that is particularly acute for TACs in neuroscience is delivery across the blood-brain barrier. These molecules are typically bulky and bifunctional, and brain exposure will strongly influence which E3 ligases and degradation pathways are practically deployable in patients. As mechanistic insight into cellular protein disposal deepens and brain-targeted delivery technologies for crossing the blood-brain barrier (for example, intranasal administration, receptor-mediated blood-brain barrier shuttles, or focused ultrasound) improve, the convergence of chemical biology and neuroscience is likely to enable next-generation therapeutics. If challenges in specificity, brain delivery, and tolerability can be overcome, this precision protein-cleanup strategy may ultimately achieve disease-modifying effects in Alzheimer’s, Parkinson’s, and related disorders.
